# Consensus for Treatment of Metastatic Castration-Sensitive Prostate Cancer: Report From the First Global Prostate Cancer Consensus Conference for Developing Countries (PCCCDC)

**DOI:** 10.1200/GO.20.00505

**Published:** 2021-04-15

**Authors:** Fernando Cotait Maluf, Felipe Moraes Toledo Pereira, Pedro Luiz Serrano Uson, Diogo Assed Bastos, Diogo Augusto Rodrigues da Rosa, Evanius Garcia Wiermann, Fábio A. Schutz, Fábio Roberto Kater, Fernando Nunes Galvão de Oliveira, Fernando Sabino Marques Monteiro, Fernando Vidigal de Pádua, Francisco Javier Orlandi, Helena Paes de Almeida Saito, Mouna Ayadi, Pamela Salman Boghikian, Ray Manneh Kopp, Ricardo Saraiva de Carvalho, Rodrigo Nogueira de Fogace, Sandro Roberto de Araújo Cavallero, Sergio Aguiar, Vinicius Carreira Souza, Silke Gillessen Sommer

**Affiliations:** ^1^ Hospital Israelita Albert Einstein, São Paulo, Brazil; ^2^ Beneficência Portuguesa de São Paulo, São Paulo, Brazil; ^3^ Latin American Oncology Group (LACOG), Rio Grande do Sul, Brazil; ^4^ Hospital São Camilo, São Paulo, Brazil; ^5^ Hospital Sirio-Libanês, São Paulo, Brazil; ^6^ Instituto do Câncer do Estado de São Paulo (ICESP), São Paulo, Brazil; ^7^ Grupo Oncoclínicas, Rio de Janeiro, Brazil; ^8^ Instituto de Oncologia do Paraná, Curitiba, Brazil; ^9^ Clinica CLION/Grupo CAM, Salvador, Bahia; ^10^ Hospital Santa Lucia, Brasília, Brazil; ^11^ Hospital Universitário de Brasília, Brasília, Brazil; ^12^ Hospital Sírio Libanês, Brasília, Brazil; ^13^ Orlandi Oncología, Santiago, Chile; ^14^ Centro de Oncologia Campinas, São Paulo, Brazil; ^15^ Institut Salah-Azaïz de Cancerologie, Tunis; ^16^ Faculté de Médecine, Tunis, Tunisia; ^17^ Fundacion Arturo Lopez Perez, Santiago, Chile; ^18^ Clínica Porto Azul, Barranquilla, Colombia; ^19^ Hospital Universitario 12 de Octubre, Madrid, Spain; ^20^ Hospital Ophir Loyol, Belém, Pará, Brazil; ^21^ Universidad de la República, Montevideo, Uruguay; ^22^ Grupo de oncologia D'Or, Rio de Janeiro, Brazil; ^23^ Oncology Institute of Southern Switzerland (IOSI), Bellinzona and Università della Svizzera Italiana, Lugano, Switzerland; ^24^ Manchester Cancer Research Centre, Division of Cancer Sciences, University of Manchester, Manchester, United Kingdom

## Abstract

**PURPOSE:**

International guideline recommendations may not always be extrapolated to developing countries where access to resources is limited. In metastatic castration-sensitive prostate cancer (mCSPC), there have been successful drug and imaging advancements that were addressed in the Prostate Cancer Consensus Conference for Developing Countries for best-practice and limited-resource scenarios.

**METHODS:**

A total of 24 out of 300 questions addressed staging, treatment, and follow-up for patients with mCSPC both in best-practice settings and resource-limited settings. Responses were compiled and presented in percentage of clinicians supporting each response. Questions had 4-8 options for response.

**RESULTS:**

Recommendations for staging in mCSPC were split but there was consensus that chest x-ray, abdominal and pelvic computed tomography, and bone scan should be used where resources are limited. In both de novo and relapsed low-volume mCSPC, orchiectomy alone in limited resources was favored and in relapsed high-volume disease, androgen deprivation therapy plus docetaxel in limited resources and androgen deprivation therapy plus abiraterone in high-resource settings were consensus. A 3-weekly regimen of docetaxel was consensus among voters. When using abiraterone, a regimen of 1,000 mg plus prednisone 5 mg/d is optimal, but in limited-resource settings, half the panel agreed that abiraterone 250 mg with fatty foods plus prednisone 5 mg/d is acceptable. The panel recommended against the use of osteoclast-targeted therapy to prevent osseous complications. There was consensus that monitoring of patients undergoing systemic treatment should only be conducted in case of prostate-specific antigen elevation or progression-suggestive symptoms.

**CONCLUSION:**

The treatment recommendations for most topics addressed differed between the best-practice setting and resource-limited setting, accentuating the need for high-quality evidence that contemplates the effect of limited resources on the management of mCSPC.

## INTRODUCTION

Prostate cancer (PCa) is the second most frequently diagnosed cancer in men worldwide, with an estimated 1.2 million new cases diagnosed in 2018, of which 80% presented with localized disease and 20% were diagnosed with an advanced or metastatic form of the disease.^1^ The burden of PCa is expected to increase to almost 2.3 million new cases and 740,000 deaths by 2040, because of growth and population aging.^1^ The existing geographical variation of PCa trends and incidence rates worldwide largely reflects the regional differences in the population distribution, with varying degrees of genetic susceptibility and access to medical care, especially in regards to the availability and use of prostate-specific antigen (PSA) screening.^2-4^

**CONTEXT**

**Key Objective**
How to find the best strategies for treatment and follow-up of patients with metastatic castration-sensitive prostate cancer (mCSPC) in the context of limited resources?
**Knowledge Generated**
This manuscript summarizes a broad consensus of clinicians from different developing countries specialized on the management of prostate cancer about optimized cost-effectives strategies in mCSPC scenario. The panel recommended more feasible staging tools such as the combination of chest x-ray, abdominal and pelvic computed tomography, and bone scan for initial approach of mCSPC and follow-up with prostate-specific antigen until clinical or biochemical progression. Besides that, panelists proposed orchiectomy alone to the treatment of low-volume mCSPC, and androgen deprivation therapy association with docetaxel or abiraterone for high-volume disease.
**Relevance**
This report provides expert recommendations to help contextualized decision making in regions of the world where international guidelines may not always be extrapolated because of limited access to resources.

The incidence of metastatic PCa at diagnosis varies widely across the globe, ranging from 5% of new PCa diagnoses in some Western countries to 60% in some areas in East Asia. Despite the relative stabilization of PCa incidence, the incidence of metastatic PCa continues to rise, with one study showing a 72% higher incidence of metastatic castration-sensitive prostate cancer (mCSPC) in the United States over the past decade.^5^ It is unclear whether this increase is related to changes in screening recommendations; however, it proves to be concerning, given that mCSPC in generally considered to be incurable.^6^ Although localized PCa has a 5-year survival rate of 100%, mCSPC has a 5-year survival rate of 29.8%.^7^ Although most patients in resource-abundant regions present with localized disease, patients in resource-limited regions tend to present with advanced disease, decreasing the possibilities of favorable treatment outcomes.^8^

mCSPC is used to describe the clinical situation whereby a patient with metastatic PCa has either never received treatment with androgen deprivation therapy (ADT) or exhibits ongoing sensitivity to ADT.^9,10^ Historically, ADT administered alone via surgical or medical castration has been the gold standard of treatment for patients with mCSPC. Both methods are equally efficacious, and the improvement in disease progression with the use of ADT has been documented extensively (32% progression in 10 years *v* 62% on the placebo group). Surgical castration performed by bilateral orchiectomy is a more cost-effective alternative to medical castration with luteinizing hormone-releasing hormone (LHRH) agonists or antagonists and may overcome healthcare access barriers and medication noncompliance that many developing countries face. However, the effect of ADT is finite and resistance to ADT occurs in most patients, resulting in progression to castrate-resistant disease, which has a median overall survival (OS) of 1-2 years.^11^

The treatment landscape in mCSPC has evolved greatly over the past 5 years, confirming that additional therapy can significantly extend a patient's progression-free and OS rate. Since 2015, two major randomized trials demonstrated the benefit of adding docetaxel chemotherapy to ADT for patients with mCSPC, with the CHAARTED trial showing an average OS 13.6 months longer and progression-free survival of 20.2 months with docetaxel compared with 11.7 months in those receiving ADT alone; the STAMPEDE trial showed an average OS of 9.76 months longer than treatment with ADT alone.^11,12^ The other alternative approved by the US Food and Drug Administration (FDA) in 2015 is the targeted therapy abiraterone acetate, supported by a branch of the STAMPEDE trial and the LATITUDE trial, which both demonstrated OS improvement to a similar degree as docetaxel.^13^ The development of these new therapeutic options in addition to ADT has improved survival outcomes and progression-free rates and is the current standard of care.^6^

In 2019, the ENZAMET and TITAN clinical trials introduced two next-generation androgen receptor inhibitors, enzalutamide and apalutamide, that have both received FDA approval for mCSPC; however, high cost and poor medical coverage of these new agents will hinder their use, especially in limited-resource settings.^14,15^

These new developments, although improving the prognosis for patients with mCSPC, present clinicians with a challenge to individualize and optimize treatment selection for each patient. There are currently no comparative data on relative efficacy on which to base selection of additional treatment; therefore, treatment considerations should include side-effect profiles, age, comorbidities, cost, and disease risk or volume.

With the incidence of mCSPC exhibiting a steady increase worldwide, clinicians and healthcare systems face several challenges in confronting the public health burden that this disease represents. This proves to be notably true in developing countries with limited resources. Although emerging therapies have expanded the treatment panorama, clinicians and governments face the challenges of balancing the adoption and availability of new treatments, their effects on quality of life and curative potential, and the cost-effectiveness and sustainability for the healthcare system.^16^

The following manuscript will summarize the recommendations of a large panel of physicians from developing countries, specializing in PCa, regarding the treatment and follow-up of patients presenting with mCSPC both with and without contemplating the restrictions of limited resources in the decision-making process, with the objective of providing guidance in clinical practice and policy development and modification. The complete methodology of Prostate Cancer Consensus Conference for Developing Countries including the elaboration process of the questionnaires to guide the panelists, the design of voting sessions, and consensus criteria were presented in the editorial and are valid for all the manuscripts.

## STAGING AND MONITORING

The imaging requirement in patients with suspected mCSPC is a modality that confirms the presence of metastases and determines their location. Current international guidelines (NCCN and European Association of Urology) do not address specific staging imaging and follow-up monitoring in patients with mCSPC because of lack of evidence.^17,18^

**FIG 1:**
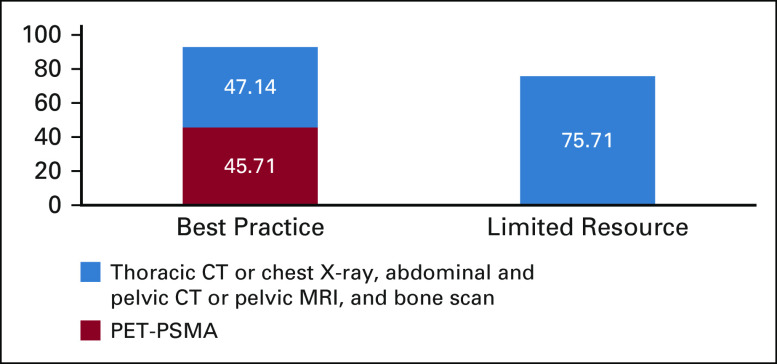
Recommended use of imaging for staging in metastatic castration-sensitive prostate cancer. CT, computed tomography; mCSPC, metastatic castration-sensitive prostate cancer; MRI, magnetic resonance imaging; PET-PSMA, positron emission tomography-prostate-specific membrane antigen imaging.

In optimal conditions, when resources are not an issue, expert opinion was split on the staging method indicated for patients with castration-sensitive prostate cancer and probable metastasis. As can be seen in Figure 1, 45.7% recommend using positron emission tomography-prostate-specific membrane antigen imaging, whereas a slight majority of 47.1% recommend using thoracic computed tomography (CT) or chest x-ray, abdominal and pelvic (or pelvic magnetic resonance imaging [MRI]), and bone scan. In settings where all imaging methods are not available, experts reached a consensus that the latter staging method should be used. Positron emission tomography-prostate-specific membrane antigen imaging for PCa is not yet FDA-approved and is considered experimental in many settings, particularly for staging of metastatic disease in addition to being more expensive.^19^ This concept is also in accordance with NCCN and European Association of Urology guidelines.^17,18^

In optimal practice settings, the vote was split on the preferred follow-up method for patients with mCSPC receiving systemic treatment, with 41.6% of the panel recommending follow-up with PSA every 3 months and thoracic, abdominal, and pelvic CT and bone scan every 6 months, and 41.6% of the panel suggesting studies should only be conducted in case of PSA elevation and/or symptoms that suggest clinical progression. The remaining physicians voted for PSA and thoracic, abdominal, and pelvic CT and bone scan every 3 months or every 6 months (13% and 3.9%, respectively).

By contrast, the panel reached consensus (81.6%) that follow-up studies in a limited-resource scenario should only be conducted in case of PSA elevation and/or symptoms suggesting clinical progression. The remainder of the panel voted for PSA every 3 months and thoracic, abdominal, and pelvic CT and bone scan every 6 months.

It is important to note that protocols for clinical follow-up have varied even between recent clinical trials. As an example, in the CHAARTED study, PSA levels were measured at each scheduled visit and imaging (CT of the abdomen and pelvis, bone scanning, and radiography or CT of the chest was performed at baseline and at the time of documented castration resistance or as clinically indicated.^11^ By contrast, in the TITAN study, patients were assessed for efficacy according to modified RECIST, version 1.1, with the use of CT or MRI of the chest, abdomen, and pelvis during screening (≤ 6 weeks before random assignment) and according to Prostate Cancer Working Group criteria^20^ with the use of bone scanning during cycles 3 and 5 and every fourth cycle thereafter.^15^ In the LATITUDE trial, efficacy assessments included sequential radiographic imaging to assess radiographic progression-free survival (CT or MRI and bone scanning) performed every 4 months, starting at week 16. PSA levels were measured at baseline, monthly in the first year, and then every 2 months until end-of-trial treatment.^13^

## TREATMENT

Combination systemic therapy is currently the standard of care for men with mCSPC. Patients should be treated with ADT via surgical (bilateral orchiectomy) or medical (LHRH agonist or antagonist) castration, in combination with a first-generation androgen receptor (AR) antagonist such as abiraterone acetate with prednisone or docetaxel (chemohormonal therapy) or with second-generation AR antagonists (enzalutamide or apalutamide), resource permitting.^17,18^ The CHAARTED study divided patients into subgroups, based on disease volume, that experienced differing survival benefit with each therapy. These criteria define high-volume disease as the presence of visceral metastases or ≥ 4 bone lesions with ≥ 1 lesion beyond the vertebral bodies and pelvis, and low-volume disease as the presence of < 4 bone lesions and absence of visceral metastases. According to the CHAARTED subgroup analysis, chemohormonal therapy may be more beneficial to men with high-volume disease, whereas abiraterone appears to be beneficial across both disease burden groups.^11^ Similar findings were seen with apalutamide and enzalutamide.^14,15^

In men with de novo mCSPC, there was no consensus across subgroups and resource settings on which hormone therapy scheme to use, as seen in Table 1. For men presenting with de novo low-volume (as defined by CHAARTED) mCSPC in the best-practice scenario, 49.3% of the physicians voted for treatment with continuous ADT by an LHRH agonist alone ± a first-generation AR antagonist and 31.5% recommended any form of continuous ADT with abiraterone for the majority of men. By contrast, when faced with the same disease in a limited-resource scenario, the majority of the panel (64.2%) recommended use of ADT by orchiectomy alone, whereas 21.4% insisted on continuous ADT by an LHRH agonist alone ± a first-generation AR antagonist. It is well known that orchiectomy is less expensive than any other option of ADT therapy. It is estimated for androgen suppression that the cost of LHRH agonist treatment is 10-13 times and combined androgen blockade is 17-20 times higher than the cost of bilateral orchiectomy (Data Supplement).^21^

**TABLE 1:**
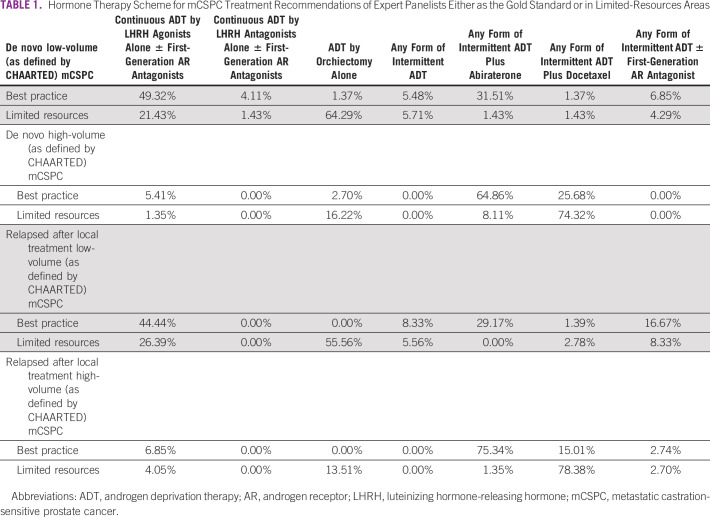
Hormone Therapy Scheme for mCSPC Treatment Recommendations of Expert Panelists Either as the Gold Standard or in Limited-Resources Areas

For de novo high-volume mCSPC (as defined by CHAARTED), 64.9% of panelists voted for any form of continuous ADT with abiraterone and 25.9% voted for any form of continuous ADT with docetaxel in a best-practice scenario. When met with limited resources, a near consensus of 74.3% of panelists recommend using ADT with docetaxel for this subgroup.^22^ This can be explained by the fact that docetaxel is far less expensive than the new hormonal therapy agents in cost-effectiveness analysis studies^23,24^ and also by a recent analysis including 566 metastatic castration-sensitive patients that did not show differences between docetaxel and abiraterone regarding overall or PCa-specific survival, nor in other important outcomes such as symptomatic skeletal events.^22^

In patients with lowvolume mCSPC who relapsed after local treatment, there was no consensus in either settings. 44.4% of the physicians polled recommended continuous ADT by LHRH agonist alone ± first-generation AR antagonist, another 29.2% recommended any form of continuous ADT with abiraterone in a best-practice setting. In a limited-resource scenario, ADT by orchiectomy alone was favored by 55.6% of the panelists, followed by continuous ADT by LHRH agonist alone ± first-generation AR antagonist with a vote of 26.4%.

It is important to note that the minority of patients included in the randomized trials comparing androgen suppression plus or minus docetaxel or abiraterone or apalutamide or enzalutamide had a recurrence after local therapy, which may explain the lack of consensus.^25^ Furthermore, another point that could add to this is the fact that relapsed metastatic disease instead of de novo metastatic disease is associated with more favorable prognosis and a distinct behavior.^26^ For example, TITAN trial included 16.4% of patients who underwent treatment for localized disease.^15^ In ENZAMET trial, about 40% of patients had prior local therapy.^14^ In LATITUDE and STAMPEDE, rates of patients with recurrent disease were lower.^12,13^

When presented with relapsed high-volume mCSPC after local treatment, there was consensus (75.3%) that any continuous form of ADT with abiraterone should be used in a best-practice setting. In a limited-resource setting, the panel convened with a vote of 78.4% that any form of continuous ADT with docetaxel should be used, with 13.5% of the panel considering ADT by orchiectomy alone the optimal choice. Table 1 summarizes physician responses to treatment recommendations in different disease burden scenarios and where therapy options may be limited because of resources.

When using castration in addition to abiraterone therapy in men with castration-sensitive disease, there was consensus (82.7%) in recommending a regimen of abiraterone 1,000 mg with prednisone 5 mg/d in best practice. 14.7% of the panel voted for a regimen of abiraterone 1,000 mg with prednisone 10 mg/d and 2.7% of the panel affirmed they do not use abiraterone in this situation. Prednisone 5 mg daily is the dose FDA-approved in association with abiraterone for high-risk metastatic castration-sensitive prostate cancer, based on LATITUDE trial.^13^

A recent randomized clinical study including 164 patients with metastatic castration-resistant prostate cancer (CRPC) compared four different regimens of corticosteroids when combined with abiraterone: once daily with prednisone, 5 mg, twice daily (n  =  41), 5 mg once daily (n  =  41), 2.5 mg twice daily (n  =  40), or dexamethasone, 0.5 mg, once daily (n  =  42). The primary end point was no mineralocorticoid excess (grade ≥ 1 hypokalemia or grade ≥ 2 hypertension) through 24 weeks (six cycles) from treatment. The conclusion of the authors was that abiraterone acetate with prednisone, 5 mg, twice daily, or dexamethasone, 0.5 mg, once daily, met the prespecified threshold for the primary end point (95% CI excluded 50% mineralocorticoid excess). By contrast, abiraterone acetate with prednisone, 5 mg, once daily, or 2.5 mg, twice daily, did not meet the threshold. Abiraterone acetate in combination with dexamethasone appeared to be particularly active but may be associated with adverse metabolic consequences.^27^

When faced with resource limitations, approximately half of the panel (52.6%) agreed on a regimen of abiraterone 250 mg with fatty foods and prednisone 5 mg/daily, whereas most of the remaining half was split between abiraterone 1,000 mg with prednisone 5 mg/daily and abstaining from using abiraterone in this situation (23.4% and 21.1%, respectively).

This result may be explained by a randomized phase II study including 72 patients with progressive CRPC that compared low-dose abiraterone (250 mg qd) with a low-fat meal versus standard-dose abiraterone (1,000 mg qd) under fasting conditions. Both arms received prednisone 5 mg twice daily. At 12 weeks, there was a greater effect on PSA in the low abiraterone arm (mean log change, −1.59) compared with standard dose (−1.19), and noninferiority of low abiraterone was established according to predefined criteria. The PSA response rate was 58% in low abiraterone and 50% in standard abiraterone arm, and the median progression-free survival was approximately 9 months in both groups.^27^

In the chemohormonal setting for men with castration-sensitive disease, there was consensus among the panel that a 3-weekly docetaxel regimen (75 mg/m^2^) should be used, regardless of resource limitations (87.7% in best-practice scenario and 97.3% in limited-resources scenario). 8.2% of panelists recommended a 2-weekly docetaxel regimen (50 mg/m^2^) in a best-practice setting.

Although the data for the 3-week schedule are more robust, a randomized trial including 177 patients with metastatic CRPC compared a 3-week (75 mg/m^2^) versus a 2-week schedule (50 mg/m^2^) and demonstrated that the latter was associated with a significantly longer time to treatment failure (5-6 months, 95% CI, 5.0 to 6.2 *v* 4.9 months, 4.5-5.4; hazard ratio 1-3, 95% CI, 1.1 to 1.6, *P* = .014) and fewer grade 3-4 adverse events such as neutropenic infections (6% *v* 24%, respectively, *P* = .002).^28^ Importantly, we have no data comparing the 2-week schedule versus the 3-week schedule in mCSPC.

Docetaxel in the 3-weekly regimen bears a relatively low risk of febrile neutropenia; however, many patients with advanced PCa present risk factors for which existing guidelines recommend that primary granulocyte-colony stimulating factor prophylaxis should be considered when starting this therapy.^17^

If chemohormonal therapy is used in men with mCSPC, there was consensus both in best-practice and limited-resource settings (75% and 93%, respectively) against the use prophylactic WBC growth factors from start of therapy in the majority of patients. In the best-practice setting, 17.3% of panelists voted in favor of this treatment. Similarly, there was overwhelming consensus across both practice settings (94.5% in best practice and 97.4% in limited resources) that prophylactic antibiotics should not be used from start of chemohormonal therapy with docetaxel. It is important to note that the standard use of G-CSF and antibiotics prophylaxis was not mandatory in neither CHAARTED nor in STAMPEDE trial.^11,12^ This information is summarized for clarity in Table 2.

**TABLE 2:**
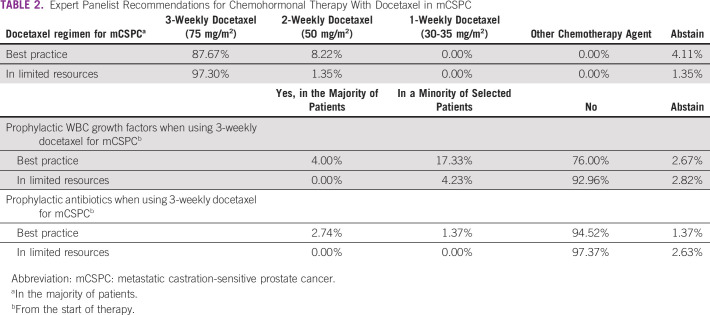
Expert Panelist Recommendations for Chemohormonal Therapy With Docetaxel in mCSPC

## OSTEOCLAST-TARGETED THERAPY FOR PREVENTION OF SKELETAL-RELATED EVENTS OR SYMPTOMATIC SKELETAL EVENTS

The majority of panelists polled affirmed that in optimal settings, they do not use osteoclast-targeted therapy for men with mCSPC with the goal of preventing bone metastasis complications, as can be seen in Table 3. 15.8% of the panelists give vitamin D and calcium supplementation alone, and the remaining 22.4% that voted on this issue recommend zoledronic acid or denosumab. For limited-resource situations, there was consensus among the panelists against using osteoclast-targeted therapy for bone metastasis complications in patients with mCSPC. 14.5% of the panel recommended vitamin D and calcium supplementation alone and the remaining 5.3% voted for zoledronic acid.

**TABLE 3:**
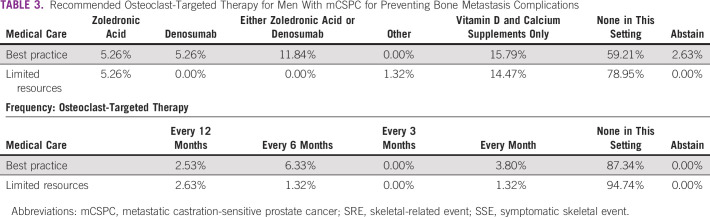
Recommended Osteoclast-Targeted Therapy for Men With mCSPC for Preventing Bone Metastasis Complications

Only in patients with metastatic castration-resistant PCa, two bone-directed agents, denosumab and zoledronic acid, have proven to prevent or delay the onset of osseous complications, with no effect on OS.^29-32^ However, NCCN guidelines do not recommend routine use of bisphosphonates or denosumab in patients with mCSPC to prevent skeletal-related events or symptomatic skeletal events during ADT, unless there is a high risk of fracture. Prevention of osteoporosis by calcium and vitamin D supplementation is recommended.^29-32^ The STAMPEDE randomized trial has not shown a benefit of OS, failure-free survival, or time to first skeletal-related events of adding zoledronic acid to castration in metastatic castration-sensitive disease.^12^ Similarly, a randomized trial including 645 patients with mCSPC compared zoledronic acid versus placebo and did not show an advantage of early zoledronic acid in increasing time to first skeletal-related event.^32^

In conclusion, this manuscript exemplifies the general practice recommendations of physicians in the field of treatment and follow-up of mCSPC in developing countries. High levels of consensus were reached in many cases regarding both best-practice and limited-resource settings, providing a practical application of expert recommendations to be available for contextualized decision making in regions of the world where international guidelines may not always be extrapolated because of limited access to resources. It should be noted that the majority of experts were Brazilian physicians, which may affect applicability to regions less represented on the panel.

In summary, the treatment recommendations for almost all the topics addressed differed between the best-practice setting and resource-limited setting, further accentuating the need for high-quality evidence that contemplates how medicine is practiced in countries facing access and resource limitations. Furthermore, although new effective treatments have become available in addition to ADT, there is little comparative data on which to base the selection of these additional treatments for men with mCSPC.
